# Biophysical and functional characterization of hippocalcin mutants responsible for human dystonia

**DOI:** 10.1093/hmg/ddx133

**Published:** 2017-04-07

**Authors:** Nordine Helassa, Svetlana V. Antonyuk, Lu-Yun Lian, Lee P. Haynes, Robert D. Burgoyne

**Affiliations:** 1 Department of Cellular and Molecular Physiology, Institute of Translational Medicine, University of Liverpool, Liverpool L69 3BX, UK; 2 Molecular Biophysics Group, Institute of Integrative Biology, Faculty of Health and Life Sciences, University of Liverpool, Liverpool L69 7ZB, UK and; 3 NMR Centre for Structural Biology, Institute of Integrative Biology, Faculty of Health and Life Sciences, University of Liverpool, Liverpool L69 7ZB, UK

## Abstract

Dystonia is a neurological movement disorder that forces the body into twisting, repetitive movements or sometimes painful abnormal postures. With the advent of next-generation sequencing technologies, the homozygous mutations T71N and A190T in the neuronal calcium sensor (NCS) hippocalcin were identified as the genetic cause of primary isolated dystonia (DYT2 dystonia). However, the effect of these mutations on the physiological role of hippocalcin has not yet been elucidated. Using a multidisciplinary approach, we demonstrated that hippocalcin oligomerises in a calcium-dependent manner and binds to voltage-gated calcium channels. Mutations T71N and A190T in hippocalcin did not affect stability, calcium-binding affinity or translocation to cellular membranes (Ca^2+^/myristoyl switch). We obtained the first crystal structure of hippocalcin and alignment with other NCS proteins showed significant variability in the orientation of the C-terminal part of the molecule, the region expected to be important for target binding. We demonstrated that the disease-causing mutations did not affect the structure of the protein, however both mutants showed a defect in oligomerisation. In addition, we observed an increased calcium influx in KCl-depolarised cells expressing mutated hippocalcin, mostly driven by N-type voltage-gated calcium channels. Our data demonstrate that the dystonia-causing mutations strongly affect hippocalcin cellular functions which suggest a central role for perturbed calcium signalling in DYT2 dystonia.

## Introduction

Dystonia is a movement disorder that causes muscle spasms and contractions. It is characterized by sustained or intermittent muscle contractions causing abnormal, often repetitive movements and painful postures than can be accompanied by dystonic tremor (1). Dystonia may be classified according to its distribution (2) or as primary or secondary (3). In contrast to secondary dystonia that can be caused by brain damage (head injury, stroke) or exposure to particular drugs, the pathophysiological mechanisms of most forms of primary dystonia are unknown (4). Recently, using a combination of homozygosity mapping and whole-exome next-generation sequencing in a consanguineous kindred affected by autosomal-recessive isolated dystonia, homozygous mutations were observed in the genes *LAPTM5*, coding for a lysosomal transmembrane protein and *HPCA*, encoding the calcium-binding protein hippocalcin. After *in silico* analysis, hippocalcin was identified as the most plausible cause for DYT2 primary isolated dystonia (5). HPCA or hippocalcin, is a member of the neuronal calcium sensor (NCS) protein family (6) and is highly expressed in hippocampal pyramidal CA1 neurons, in particular in their dendrites (7–9). It contains four EF-hands out of which only three are capable of binding calcium. In response to increased intracellular calcium, hippocalcin undergoes a conformational change and translocates from the cytosol to cellular membranes through a Ca^2+^/myristoyl switch mechanism (10). The extrusion of the myristoyl-containing hydrophobic N-terminus region from the hydrophobic pocket (11) allows hippocalcin to localize to membranes where it can interact with downstream targets (10,12,13). In addition to its role in the control of apoptosis (14), hippocalcin has been shown to be involved in neuronal excitability (15–17), regulation of neurite outgrowth (18) and gene transcription (19,20), long-term depression (21–23) and the modulation of cyclic nucleotide signalling (24). Mutations at positions T71N and A190T were shown to be critical in development of DYT2 dystonia (5). It was speculated that T71N mutation could impair or prevent calcium binding to EF-hand domain 2, possibly leading to a loss of function, whereas A190T could be involved in target specificity. However, no direct evidence was provided to support these hypotheses.

In this study, we investigate the effect of the dystonia-causing mutations on the biophysical and physiological properties of hippocalcin. We demonstrate that hippocalcin oligomerises upon calcium binding and interacts with voltage-gated calcium channels (VGCCs). We observed that the structure, stability and calcium-binding properties of the mutants remain unchanged. Both mutants, however, show strong oligomerisation defects and increased intracellular calcium influx. These results suggest that the cause of the disease is not due to loss of hippocalcin expression or stability but to subtle changes in its ability to participate in calcium signalling.

## Results

### Calcium binding and stability properties of hippocalcin-mutant proteins remain unchanged

**Figure 1:**
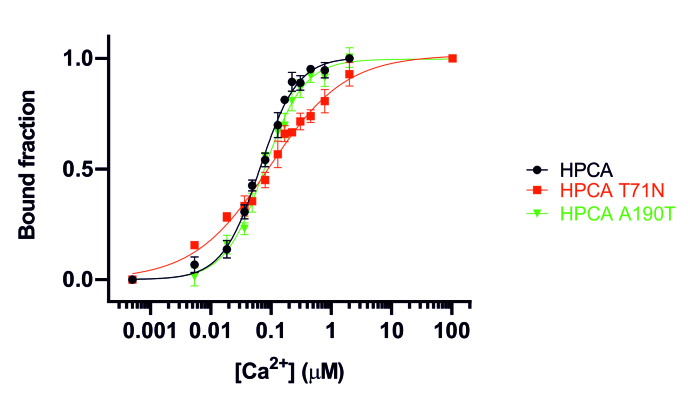
Equilibrium Ca^2+^-binding titrations for hippocalcin wild-type and mutants measured by intrinsic tryptophan fluorescence at 20 °C. Fluorescence changes (λ_ex_ = 280 nm and λ_em_ = 340 nm) are normalised to *F*_0_ of 0 and *F*_max_ of 1 and fitted to the Hill equation. Fitted curves are represented by solid lines overlaying the data points. Titrations were performed at least in triplicates and represented as mean ± SEM.

To determine if the calcium-binding properties of hippocalcin were altered by the mutations, equilibrium calcium-binding titrations were performed using intrinsic tryptophan fluorescence (Fig. 1). Free calcium concentrations ([Ca^2+^]) were calculated using the Maxchelator program (25) and were verified using OGB-1 (Supplementary Material, Fig. S1). In order to determine the dissociation constant (*K*_d_) and the Hill coefficient (*n*), calcium titrations were normalised and fitted to the Hill equation (correlation coefficient values between 0.96 and 0.98). Hippocalcin wild-type had a *K*_d_ of 65 ± 4 nM and a Hill coefficient (*n*) of 1.4 ± 0.1. As expected, hippocalcin(A190T) presented similar calcium-binding properties to the wild-type protein with *K*_d_ = 77 ± 5 nM and *n* = 1.3 ± 0.1 (Table 1). Affinities for calcium for hippocalcin(T71N) (*K*_d_ = 93 ± 12 nM) was not significantly different from the wild-type, even though the mutation was located within the second EF-hand of hippocalcin. However, the cooperativity of calcium binding, shown by the Hill coefficient, significantly decreased from *n* = 1.4 ± 0.1 for the wild-type, to *n* = 0.7 ± 0.1 for hippocalcin(T71N).

**Table 1:** Calcium-binding properties of HPCA wild-type and disease-causing mutants

	*K*_d_ (nM)	*n*	*R*^2^
OGB-1	132 ± 11	1	0.97
HPCA	65 ± 4	1.4 ± 0.1	0.98
HPCA T71N	93 ± 12	0.7 ± 0.1*	0.96
HPCA A190T	77 ± 5	1.3 ± 0.1	0.97

* *P* < 0.01, compared to HPCA wild-type, student *t*-test.

Stability of hippocalcin proteins was determined by trypsin limited proteolysis and temperature/chemical denaturation (Supplementary Material, Fig. S2). Incubation of wild-type hippocalcin with trypsin showed 32 ± 14% degradation over 30 min (Supplementary Material, Fig. S2A). Similar susceptibility to protease degradation was observed for both mutants. In addition, α-helices temperature unfolding followed by circular dichroism revealed that hippocalcin is a very thermostable protein with only 37% of α-helices denaturation at 90 °C, and that the mutations did not alter the temperature stability of the protein (Supplementary Material, Fig. S2B). Guanidine denaturation monitored by intrinsic tryptophan fluorescence showed complete protein denaturation at [Gnd-HCl] of 3 M (Supplementary Material, Fig. S2C). When comparing the susceptibility to guanidine of hippocalcin wild-type with the mutants, no significant difference was observed. Altogether, these data suggest that the disease-causing mutations T71N and A190T are not affecting the stability of the proteins.

### Disease-causing mutants have a similar secondary and 3D structure to the parent protein

Circular dichroism (CD) spectroscopy was used to examine whether the mutations in hippocalcin were causing secondary structure modifications. CD spectra were collected for hippocalcin and the mutants in either apo- or calcium-bound form (Supplementary Material, Fig. S3A). After protein secondary structure prediction using the CDSSTR method, the helical content of hippocalcin was estimated to ∼75% of α-helices and only 9% of β-sheets (Supplementary Material, Fig. S3B). The conformational change that occurs upon calcium binding did not affect the secondary structure content, neither did the mutations.

**Figure 2:**
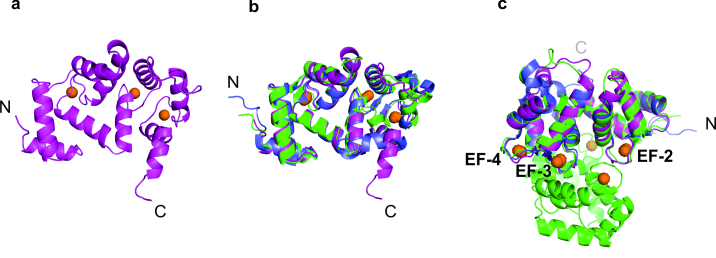
Cartoon representation of the crystal structure of the wild-type hippocalcin and its comparison with the structures of other calcium-binding proteins. (**A**) Wild-type human hippocalcin (magenta) with 3 Ca^2+^ ions (orange spheres) bound at EF-2, EF-3 and EF-4 sites. Alignment of human hippocalcin crystal structure (magenta) with (**B**) rat NCS-1 (blue) (PDB 5AEQ) and bovine neurocalcin delta (green) (PDB 1BJF), revealing a different orientation of the C-terminal region of the molecule; (**C**) rat NCS-1 (blue) (PDB 5AEQ) and dimeric bovine neurocalcin delta (green) (PDB 1BJF), showing structural conservation of the EF-hands, located at the dimerization interface.

Crystal structures were obtained for wild-type human hippocalcin (PDB 5G4P), hippocalcin(T71N) (PDB 5M6C) and hippocalcin(A190T) (PDB 5G58) at a resolution of the 2.42, 3.00 and 2.54 Å, respectively. Data collection and refinement statistics are presented in Supplementary Material, Table S1. The structure of hippocalcin is similar to all NCS proteins and contains 4 EF-hands (helix-loop-helix motifs) and 10 α-helices (Supplementary Material, Fig. S4A). Electron density revealed three calcium ions in EF-2, EF-3 and EF-4 binding sites (Fig. 2A) in all structures. Cryptic EF-hand 1 did not bind any calcium ions, as expected. The regions involved in calcium binding showed complete preservation and high order. As many NCS proteins, hippocalcin has two lobes, N-lobe is formed by EF-1 and EF-2, while C-lobe is formed by EF-3 and EF-4. The crystal structure contained two copies of hippocalcin in the asymmetric unit connected by insertion of C-terminal helix of chain E (residues 181–190) into the hydrophobic groove of chain A. This packing is facilitated by salt bridge between guanidinium group of Arg181 and carboxyl group of Asp37, and hydrogen bond between OE1 Gln184 and NE1 Trp30 (Supplementary Material, Fig. S4B). Interestingly, the same fragment is expected to be involved in target binding. PISA analysis of the oligomeric states for all three proteins has confirmed that they are monomeric in solution. Alignment of Cα atoms of hippocalcin with other calcium-binding proteins such as rat NCS-1 (PDB 5AEQ) and bovine neurocalcin delta (PDB 1BJF) showed significant variability in the orientation of the C-terminal part of the molecule, region expected to be involved in specificity of target binding (Fig. 2B). In addition, significant variability was observed in the loop regions, with exception of the EF-hand loops (Fig. 2C). Comparison of hippocalcin with the dimeric crystal structure of bovine neurocalcin delta (PDB 1BJF), suggest a key role of the calcium EF-hands for oligomerisation as they seem to be located at the dimerization interface and are structurally identical in 3 proteins (Fig. 2C). The crystal structure of hippocalcin(T71N) and hippocalcin(A190T) did not show any significant conformation difference when compared to the wild-type protein and they have identical crystal packing (Supplementary Material, Fig. S4C). Mutation site in hippocalcin T71N is located on the interface between N-lobe and C-lobe close to the area of interaction with peptides in NCS-1 (26), change from Thr to Asn strengthens inter-lobe interface by creating additional hydrogen bond between ND2 Asn71 and OG Ser106 (Supplementary Material, Fig. S4D). Mutation A190T is located at the end of the flexible C-terminal helix (Supplementary Material, Fig. S4E), which is predicted to interact with hippocalcin target protein. Change from Ala to Thr does not decrease C-terminus flexibility.This part has no electron density in chain A.

### Hippocalcin forms multimers in the presence of calcium and disease-causing mutants show a strong oligomerisation defect

**Figure 3:**
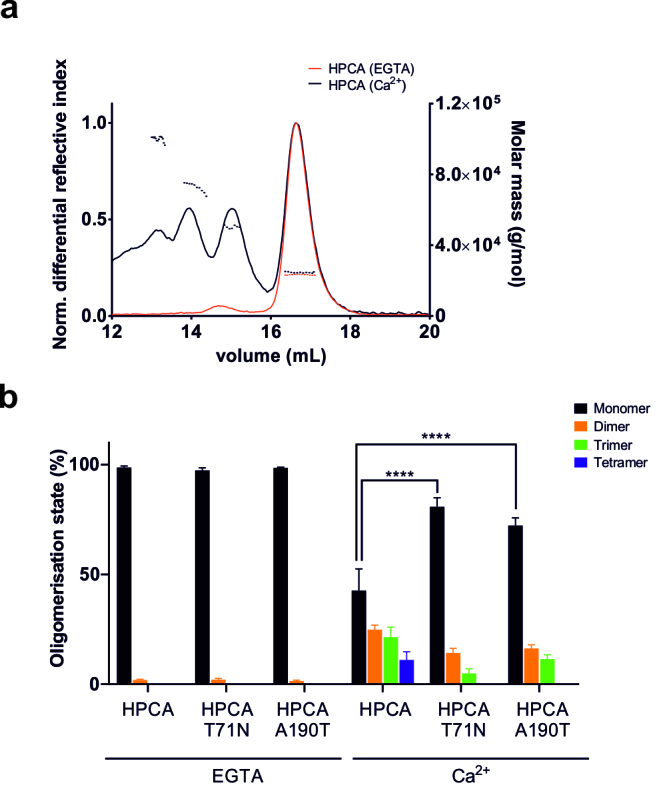
Defect of oligomerisation for hippocalcin mutants measured by SEC-MALS. (**A**) Representative SEC-MALS data obtained for hippocalcin wild-type showing a monodisperse sample in the presence of 5 mM EGTA (red) (consistent with a monomer) compared to a polydisperse sample in the presence of 1 mM CaCl_2_ (black) (consistent with a mixture of monomer, dimer, trimer and tetramer). The average molar mass is indicated in dotted line and the differential refractive index in plain line. (**B**) Analysis of the MALS data for hippocalcin wild-type and mutants in the presence or absence of Ca^2+^. Molar mass of each peak is shown in Supplementary Material, Table S2.

Absolute molar masses of hippocalcin wild-type and mutants were accurately determined by SEC-MALS, overcoming the limitation of column calibration. MALS data indicated that in the absence of calcium, hippocalcin was 99% monomeric (23.1 ± 0.2 kDa) (Fig. 3, Supplementary Material, Table S2). However, in the presence of 1 mM calcium, hippocalcin formed a mixture of monomers (25.1 ± 0.9 kDa) and oligomers with a molar mass consistent with dimer (46.7 ± 1.1 kDa), trimer (68.2 ± 1.9 kDa) and tetramer (91.6 ± 2.9 kDa) molecules. The oligomeric fraction represented more than 57% of the total proteins. As with the parent protein hippocalcin, the hippocalcin(T71N) and hippocalcin(A190T) proteins were monodisperse in the absence of calcium with a size consistent with a monomer (23.2 ± 0.2 kDa on average) and formed oligomers in a calcium-dependent manner (Fig. 3B). The calcium-dependent oligomerisation was also observed in *in vitro* crosslink (BS3 or DSP) experiments (Supplementary Material, Fig. S5A–C). Indeed, densitometry quantification after DSP crosslinking on hippocalcin purified protein showed that calcium significantly increased the polydispersity of the sample (from 24 ± 3% oligomers in the absence of calcium, to 52 ± 1% oligomers in the presence of calcium). Treatment with DTT after DSP crosslinking cleaved the oligomers back into monomers, as expected (Supplementary Material, Fig. S5B). Oligomerisation also occurred at low calcium concentrations (50 μM) (Supplementary Material, Fig. S5C). Intracellular crosslink in N2A cells using DSP reproduced the calcium-dependent oligomerisation of hippocalcin, in a physiological context (Supplementary Material, Fig. S5D).

Oligomerisation upon calcium binding was also observed for the disease-causing mutants hippocalcin(T71N) and hippocalcin(A190T) (Fig. 3B, Supplementary Material, Fig. S5A,B,D). However, the mutations significantly decreased the oligomer formation when compared to the wild-type protein (Supplementary Material, Fig. S5B). Indeed, the fraction of oligomers measured by MALS was reduced from 57% for the wild-type protein to 19–28% for the mutants (Fig. 3B). Using a combination of biochemical and biophysical techniques, we demonstrated the calcium-dependent oligomerisation of hippocalcin and a strong defect in oligomer formation for the dystonia-causing mutants.

### Hippocalcin and mutants bind to P/Q- and N-type voltage-gated calcium channels (VGCCs)

**Figure 4:**
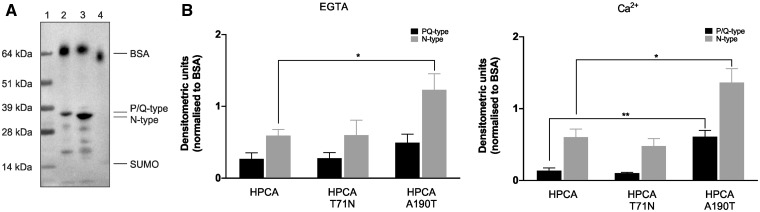
Hippocalcin wild-type and mutants interact with P/Q- and N-type voltage-gated calcium channels. P/Q- (residues 1898-2035), N-type (residues 1836-1983) VGCC fragments and SUMO protein were separated by SDS-PAGE and transferred to nitrocellulose. Binding proteins were detected with biotinylated hippocalcin or hippocalcin mutants in the absence or presence of Ca^2+^. (**A**) Representative overlay blot after detection with streptavidin-HRP. Lane 1: molecular weight ladder, lane 2: SUMO-P/Q- purified protein, lane 3: SUMO-N-type purified protein, Lane 4: SUMO purified protein. Biotinylated BSA was added to the each sample and used as a loading control. (**B**) Quantification of hippocalcin binding to the P/Q- and N-type VGCC fragments using ImageJ showed calcium-dependent altered binding of A190T mutant to both P/Q- and N-type channels compared to the wild-type protein. All experiments were performed in 3-6 replicates and expressed as normalised mean ± SEM.

Hippocalcin wild-type has been shown to affect cellular response to membrane depolarisation and it has been suggested that hippocalcin might play a role in regulating voltage-dependent calcium channels (5). However, there is yet no direct evidence of hippocalcin interacting with VGCCs. In this study, we investigate the interaction of hippocalcin with P/Q- and N-type channels as other NCS family members have been shown to bind to these channel subtypes (27–29), thought to be drivers of synaptic transmission (30). We demonstrate, using purified proteins and western blots, that hippocalcin wild-type and mutants bound to both P/Q- (residues 1898-2035) and N-type (residues 1836-1983) VGCC fragment proteins whereas no significant binding to the SUMO protein control was detected (Fig. 4A). In addition, after densitometry quantification using biotinylated BSA as loading control, we observed that both mutants also bound to the channel fragments and that hippocalcin(A190T) interaction with VGCCs was altered (Fig. 4B). Indeed in the absence of calcium, hippocalcin(A190T) presented increased interaction with N-type channels when compared to the wild-type. In the presence of low μM calcium hippocalcin(A190T) showed significantly enhanced binding to both P/Q- and N-type calcium channels, compared to the parent protein. We have been unable to confirm this interaction for native channel subunits using immunoprecipation techniques as an effective anti-hippocalcin antiserum is currently not available.

### Functional characterisation of hippocalcin wild-type and mutants

**Figure 5:**
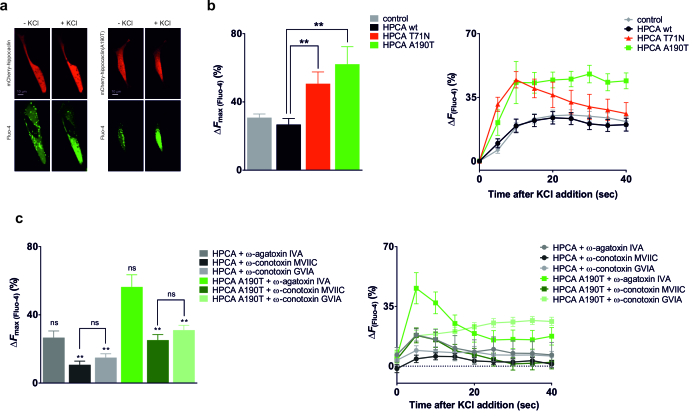
Dystonia-causing hippocalcin mutants increase depolarisation-induced calcium influx through N-type voltage-gated calcium channels. Differentiated SH-SY5Y cells transfected with hippocalcin-mCherry constructs were loaded with Fluo-4 to monitor calcium concentration changes. After KCl depolarisation, live cells were imaged on a spinning-disk confocal microscope. (**A**) Representative microscopy images of KCl-stimulated cells transfected with hippocalcin or hippocalcin(A190T), showing intracellular calcium increase upon depolarisation. (**B**) Maximum intracellular calcium increase and time course after KCl stimulation, showing that hippocalcin(T71N) and hippocalcin(A190T) increases calcium entry in response to depolarisation. Transfection control (*n =* 15), hippocalcin wild-type (*n =* 13), hippocalcin(T71N) (*n =* 15) and hippocalcin(A190T) (*n =* 14). Data are expressed as mean ± SEM. Statistical analysis was performed using hippocalcin as control (Student *t*-test). (**C**) Maximum intracellular calcium increase and time course after KCl stimulation, showing that calcium entry in response to depolarisation is determined by N-type voltage-gated calcium channels. Hippocalcin wild-type + 0.4 μM ω-agatoxin IVA (*n =* 9), hippocalcin wild-type + 10 μM ω-conotoxin MVIIC (*n =* 10), hippocalcin wild-type + 1 μM ω-conotoxin GVIA (*n =* 13), hippocalcin(A190T) + 0.4 μM ω-agatoxin IVA (*n =* 14), hippocalcin(A190T) + 10 μM ω-conotoxin MVIIC (*n =* 15), hippocalcin(A190T) + 1 μM ω-conotoxin GVIA (*n =* 22). Data are expressed as mean ± SEM. Statistical analysis was performed using untreated hippocalcin or untreated hippocalcin(A190T) as controls (Student *t*-test).

Differentiated SH-SY5Y cells were transfected with mCherry-tagged hippocalcin constructs and loaded with Fluo-4 calcium dye. Expression levels were verified by Western blot using actin, calnexin and tubulin as loading controls and did not show any difference between the wild-type and the mutants (Supplementary Material, Fig. S6). After 50 mM KCl stimulation to depolarise the cells, calcium signals were monitored on a spinning-disk confocal microscope (Fig. 5). Cells transfected with hippocalcin wild-type showed 27% increase in intracellular fluorescence after KCl depolarisation. For the dystonia-causing mutants hippocalcin(T71N) and hippocalcin(A190T), calcium influx upon stimulation significantly increased to 51% and 62%, respectively, compared to hippocalcin wild-type (Fig. 5B). We set out to determine which type of calcium channel mediated the increased calcium influx by testing the effect of calcium channel blockers on cells expressing wild type hippocalcin or hippocalcin(A190T). For both hippocalcin- and hippocalcin(A190T)-transfected cells, incubation with ω-agatoxin IVA (P/Q-type channel blocker) did not significantly affect calcium influx upon KCl-induced depolarisation (Fig. 5C). However, we observed a large decrease of the calcium response in the presence of ω-conotoxin MVIIC (P/Q- and N-type channel blocker) and ω-conotoxin GVIA (N-type channel blocker). For both hippocalcin and hippocalcin(A190T), fluorescence calcium signal decreased by ∼60% in the presence of ω-conotoxin MVIIC and ∼50% in the presence of ω-conotoxin GVIA. These results suggest that the increased calcium response due to hippocalcin(A190T) expression was predominantly due to calcium entry through N-type calcium channels.

After rapid calcium photorelease, hippocalcin translocation to the preinuclear region of the cells was observed (Supplementary Material, Fig. S7A). This was, as previously demonstrated by O’Callaghan and coworkers, to be due to translocation to the *trans*-Golgi network (TGN) (10,31). Time-courses of Fluo-4 (calcium indicator) and hippocalcin fluorescence revealed that translocation is calcium-dependent and that it occurred rapidly after calcium rise (Supplementary Material, Fig. S7B). Photocleavage of NP-EGTA to rapidly release intracellular calcium also induced translocation of hippocalcin(T71N) and hippocalcin(A190T) mutants to the TGN.

## Discussion

Dysregulation in calcium homeostasis and signalling have been implicated in many aspects of neuropathology, neurodegeneration and psychiatric disorders (32–36). In this paper, we investigated the role of mutations in hippocalcin, a neuronal calcium sensor involved in DYT2 dystonia disease. Using a combination of biophysical, structural biology and cell biology techniques, we demonstrated that the disease-causing mutations T71N and A190T have an effect on key functional properties of hippocalcin.

In many cases disease-causing mutations can affect protein folding and/or reduce protein stability leading to a loss of expression levels. This was not the case for the DYT2 hippocalcin mutations. Biophysical experiments showed that all hippocalcin mutants had similar CD spectrum with no altered protein stability when compared to the wild-type protein. Heat-denaturation studies revealed that hippocalcin is a very thermostable protein, as observed for other calcium-binding protein such as VILIP-1 with unfolding temperature *T*_m_ > 110 °C (37), Calmodulin and Troponin C (*T*_m_ > 90 °C) (38). The Ca^2+^-binding data were performed using unmyristoylated hippocalcin. There is no evidence available on whether myristoylation affects the Ca^2+^ binding to hippocalcin. While we cannot exclude minor effects, analysis of the unmyristoylated hippocalcin proteins should allow us to detect any differences in the intrinsic Ca^2+^ affinity of the wild-type compared to the mutants. Equilibrium calcium-binding titrations showed that hippocalcin has a high affinity for calcium binding (*K*_d_ = 65 nM) with positive cooperativity (*n* = 1.4) as observed for other NCS proteins such as VILIP-1 (37), recoverin (39,40) and neurocalcin delta (39,41). Surprisingly, mutation hippocalcin(T71N), located within the second EF-hand, did not alter the overall calcium- binding affinity but significantly reduced the calcium-binding cooperativity. Previous studies on VILIP-1 and recoverin revealed that two calcium ions bind sequentially, first at EF-3 (high affinity) to facilitate cooperative calcium binding at EF-2 (low affinity) (37,40). Because EF-2 has low affinity for calcium (μM), mutations in this domain in hippocalcin may not have any significant effect on the overall dissociation constant. The equilibrium calcium-binding titrations for hippocalcin and hippocalcin(A190T) show that a calcium concentration of ∼1 μM is sufficient to get the protein fully Ca^2+^-bound. However, the decrease in cooperativity observed for hippocalcin(T71N) suggests that at low μM [Ca^2+^], hippocalcin(T71N) will only be partially Ca^2+^-bound which then could affect regulation of downstream calcium signalling cascades in stimulated cells.

Hippocalcin shares common structural features of NCS proteins of ∼200 residue chain containing 4 EF-hand motifs and an N-terminal myristoylation consensus sequence. The three dimensional structures are known for NCS-1 (42,43), recoverin (11,44) and neurocalcin delta (45). Here we provide the first crystal structure for calcium-bound hippocalcin, hippocalcin(T71N) and hippocalcin(A190T) with a resolution of 2.4–3.0 Å. There is no significant difference between the three structures. However, comparison of hippocalcin crystal structure with neurocalcin delta and NCS-1 revealed a different orientation of the C-terminus part of the protein. In NCS-1 the C-terminal tail appears to be able to sit in the hydrophobic groove in the absence of ligand and there is evidence to show that this tail acts as an auto-regulatory mechanism to control target binding (46–48); it is not clear at this stage if the C-terminal tail of hippocalcin serves the same function but it also sits in the hydrophobic groove in observed crystal packing (Supplementary Material, Fig. S4C).

We demonstrated that hippocalcin oligomerises upon calcium binding with molar masses consistent with dimers, trimers and tetramers. We showed by *in vitro* crosslinking experiments that 50 μM Ca^2+^ were sufficient to trigger oligomer formation. In addition, we demonstrated that hippocalcin oligomerises in cells stimulated with ionomycin, which raises the intracellular calcium to ∼1 μM (49,50). Calcium concentration at a mouth of an open calcium channel is predicted to be tens to hundreds of micromolar (51,52), suggesting that hippocalcin may bind to the channel as an oligomer. VILIP-1 and neurocalcin delta, neuronal calcium sensors from the same family, also forms an equilibrium mixture of monomeric and dimeric protein species (37,53,54), in contrast to NCS-1 which is a monomeric protein (26). Disease-causing mutants of hippocalcin showed a marked oligomerisation defect suggesting that EF-2 and the C-terminus domain are important for dimerization. In addition, crystal structure of dimeric neurocalcin delta (PDB 1BJF) suggest an important role of the EF-hands in the oligomerisation of the protein, which we cannot confirm from our crystal structures.

Upon intracellular calcium elevation, calcium sensor proteins undergo a conformational change enabling interactions with partners and regulation of various calcium signalling pathways (28). Hippocalcin exhibits a dynamic calcium association in response to calcium increase and neuronal activity through a Ca^2+^/myristoyl switch mechanism (11). The change in conformation induces its binding to cellular membranes where it can interact with target proteins (10–12,55). Even though the bulk of hippocalcin becomes translocated to the TGN, a proportion also translocates to the plasma membrane (10,31), where it can interact with VGGCs. The dystonia-causing mutation T71N and A190T did not alter the translocation process.

Entry of extracellular calcium into synaptic terminals through VGCCs is the driving force for exocytosis of neurotransmitter-containing vesicles. Hippocalcin has been implicated as a calcium sensor in long-term depression and the gating of channels underlying a slow after-hyperpolarisation current (16,21,56). However, no direct interaction with VGCCs channels has been shown. In this paper, we demonstrated that hippocalcin directly binds to the α-subunit of VGCCs of P/Q- and N-type, which suggest a role of hippocalcin in neurotransmitter release and synaptic plasticity. Hippocalcin(A190T) showed increased binding to both channels compared to the wild-type protein, suggesting that the mutation in the C-terminus region is important for target interaction, as previously shown for other NCS proteins (46,57). We demonstrated that hippocalcin can bind to the VGCCs in the absence of calcium, suggesting a pre-association to the pore-forming α-subunit of VGCCs as observed for apo-CaM (58–60). In cells transfected with hippocalcin mutants, we observed a significant increase in the calcium response to KCl depolarisation, compared to wild-type hippocalcin. Application of N-type calcium channel blockers (ω-conotoxins MVIIC and GVIA) dramatically reduced calcium influx and the increased calcium signal due to hippocalcin(A190T), whereas the P/Q channel blocker ω-agatoxin IVA had no effect. These findings suggest that the effect of hippocalcin mutants on calcium signalling is driven by interaction and regulation of N-type calcium channels. Similarly, other calcium-binding proteins such as CaM, CaBP1 and the NCS family members NCS-1 and VILIP-2 have been reported to associate with the α-subunit cytoplasmic regulatory domain of VGCCs of P/Q-, N- and L-type to modulate channel opening and therefore synaptic plasticity (27–29,61).

The binding to VGCCs of the mutants and the increased intracellular calcium influx in stimulated cells could contribute to a dysregulation of subsequent signalling pathways and result in dramatic physiological alterations.

In summary, we demonstrated that hippocalcin forms oligomers upon calcium binding and directly interacts with VGCCs. The dystonia-causing mutations did not affect protein stability or folding. In common for both T71N and A190T mutants was an impaired calcium-dependent oligomerisation and increased intracellular calcium influx after KCl depolarisation. These functional alterations suggest that the observed defect in hippocalcin-dependent calcium signalling would have important physiological consequences that contribute to the onset of the disease.

## Materials and Methods

### Plasmids

A series of site-directed mutations were performed on pHippo-EYFP expressing human hippocalcin following the QuikChange protocol (Agilent Technologies) to generate pHPCA-EYFP constructs using the following primers: T71N, 5’-CGAGCATGTCTTCCGCAATTTTGACACCAACAGCG-3’; A190T, 5’-GCGATCCCAGCAGCACTTCCCAGTTCTCG-3’; and confirmed by DNA sequencing (DNA sequencing and Services, University of Dundee). For multi-color imaging, hippocalcin genes were subcloned from pHPCA-EYFP into pEGFP-N1 or pmCherry-N1 vectors by restriction-ligation (NheI/XmaI), generating pHPCA-EGFP and pHPCA-mCherry constructs. For protein expression and purification, hippocalcin genes were amplified by PCR from pHPCA-EYFP vectors using the following primers: sense, 5’-CTATGAATTCATGGGCAAGCAGAATAGC-3’; antisense: 5’-CTATCTCGAGTCAGAACTGGGAAGCGCTGCTG-3’, and cloned by restriction-ligation into pGEX-6P1 (EcoRI/XhoI). Residues 1898-2035 from rat P/Q-type calcium channel α−subunit (NM_012918) were amplified by PCR from GST-PQ (62) using the following primers: sense, 5’-GGTCTCTAGGTAAGTCCACGGCCTGACAG-3’; antisense, 5’-ATATCTCGAGCTAGGGGAGGTAGTGTTCGCT-3’; and subcloned into pE-SUMO by restriction-ligation (BsaI/XhoI). DNA fragment encoding residues 1836-1983 from mouse N-type calcium channel α−subunit (NM_001042528) were obtained from GeneArt (Thermo Fisher Scientific) using the following primers: sense, 5’-GGTCTCTAGGTAAACCTGACGAGATGACAG-3’; antisense, 5’-ATATCTCGAGCTACCCAGGCTGGGGCTCCCC-3’; and cloned into pE-SUMO by restriction-ligation (BsaI/XhoI). The P/Q- fragment was chosen according to previous work (62) and the N-type fragment was designed as the region with greatest sequence homology to the P/Q- fragment.

### Cell culture and transfection

Cells were cultured in DMEM containing non-essential amino-acids (Life Technologies), penicillin/streptomycin (100 U/ml, 100 μg/ml, respectively), at 37 °C in an atmosphere of 5% CO_2_ in. Mouse N2A neuroblastoma culture medium was supplemented with 5% Heat inactivated FBS (Life Technologies) whereas SH-SY5Y neuroblastoma cells were cultured in DMEM/F-12 supplemented with 10% Heat inactivated FBS (Life Technologies). SH-SY5Y neuroblastoma cells were differentiated in DMEM/F-12 supplemented with 1% Heat inactivated FBS (Life Technologies) and 10 μM retinoic acid for 3–5 days. Cells were plated on 35-mm glass bottom culture dishes (MatTek) and allowed 24 h to adhere before transfection with FuGENE HD (Promega) following the manufacturer’s recommendations. Cells were maintained for 12–24 h before being used in experiments.

### Expression and purification of hippocalcin and VGCCs fragment proteins

*Hippocalcin.* Hippocalcin wild-type and mutant proteins were overexpressed in *E. coli* BL21(DE3) STAR cells overnight at 20 °C after addition of 0.5 mM IPTG. Cells were resuspended in 50 mM Na^+^-HEPES, 200 mM NaCl, pH 7.5 containing EDTA-free Complete protease inhibitor cocktail (Roche) and lysed by 1-h incubation with 1 mg/ml of lysosyme and sonication on ice (VibraCell, Jencons PLS). Lysates were incubated with benzonase 1 h on ice and clarified by ultra-centrifugation at 100,000g. Clarified lysates were purified using 5 ml of GST resin (GST agarose, ThermoFisher Scientific) equilibrated with 50 mM Na^+^-HEPES, 200 mM NaCl and GST-tag was cleaved using the PreScission protease. The purified cleaved protein was further purified on an AKTA Purifier using a Superdex 200 (26/60) gel filtration column equilibrated with 50 mM Tris pH 7.5. Elution fractions were concentrated on Centricon 10 kDa and aliquots were stored at −80 °C.

*VGCCs.* SUMO, SUMO-P/Q- and SUMO-N-type fragment proteins were expressed as described above. Clarified lysates were purified using 1 ml of Ni-NTA resin (QIAGEN) equilibrated with 50 mM Na^+^-HEPES, 200 mM NaCl and eluted with 500 mM imidazole. Fractions were aliquoted and stored at −80 °C.

Purity of the eluted fractions was determined by SDS-PAGE (NuPAGE 4–12% Bis-Tris, NuPAGE MOPS SDS running buffer, Life Technologies) and concentration was measured by spectrophotometry at 280 nm (ε_0 (hippocalcin)_ = 20 065 M^−1^ cm^−1^), (ε_0 (P/Q)_ = 27 515 M^−1^ cm^−1^), (ε_0 (N)_ = 28 028 M^−1^ cm^−1^), (ε_0 (SUMO)_ = 12 010 M^−1^ cm^−1^) (Nanodrop Lite, Thermo Scientific). Molar extinction coefficient was calculated from the amino acid composition using ExPASy/ProtParam program (10,63).

### Biotinylation of hippocalcin proteins

Hippocalcin proteins or BSA (200 μg/ml = 10 μM) were incubated with 100-fold molar excess of 6-(biotinamidocaproyamindo) N-hydroxysuccimide ester (Sigma) for 2 h at room temperature. Excess biotin reagent was removed by extensive dialysis against 20 mM Na^+^-HEPES, 100 mM NaCl, pH 7.5. Biotinylated proteins were aliquoted and stored at −80 °C.

### Equilibrium Ca^2+^ binding using tryptophan fluorescence

Hippocalcin proteins (1 μM) were titrated with increasing CaCl_2_ concentrations in 50 mM K^+^-HEPES, 100 mM KCl, 2 mM MgCl_2_, 5 mM EGTA, pH 7.5. Intrinsic fluorescence was measured at 280 nm excitation and 340 nm emission wavelengths in a 1 ml quartz cuvette using a JASCO FP-6300 spectrofluorimeter (Jasco) at 20 °C. All titrations were performed at least in triplicates and expressed as mean ± SEM. Data were corrected for dilution, normalised and Ca^2+^ dissociation constant (*K*_d_) and cooperativity (*n*) were obtained by fitting to the Hill equation (Prism GraphPad 6). [Ca^2+^] were calculated using the two-chelators Maxchelator program (25) and verified by OGB-1 titration. Data were fitted to a one-site specific binding equation using Prism GraphPad 6 software, giving a *K*_d_ for OGB-1 for Ca^2+^ of 132 ± 11 nM, similar to the value reported by Molecular Probes (*K*_d_ = 170 nM, in the absence of Mg^2+^).

### Structure determination of hippocalcin proteins

Crystals for hippocalcin, hippocalcin(T71N) and hippocalcin (A190T) proteins were grown at 20 °C using the sitting drop vapour diffusion method at a concentration of 50 mg/ml. Wild-type protein crystallised in 0.1 M sodium citrate tribasic dihydrate pH 5.5, 18% v/v 2-propanol, 16% w/v PEG 4000. Hippocalcin(A190T) crystallised in the same condition but with 20% w/v PEG 4000. Crystals were cryo-protected using paratone-N and diffraction data were collected at Diamond synchrotron, beamlines IO2, IO4-1 and IO4, data were processed by iMOSFLM (64) and scaled by Aimless (65). Structure for wild-type protein was solved by molecular replacement with MOLREP (66), using NCS-1 (PDB 5AEQ) as a search model, structures of mutants were refined starting from wild-type structure using REFMAC5 (67) in the CCP4 (68) programme suite with isotropic B-factors, NCS restrains. TLS refinement was used during the last stages. Rebuilding of the model between refinement cycles and adding water molecules was performed in COOT (69). The quality of the models was assessed on MolProbity (70) server. For wild-type protein, residues from 6-188/7-189 for A190T mutant from 6-189/7-190; for T71N mutant from 6-188/7-190, were clearly visible for both chains A and E, respectively, in spite of high Wilson B-factor for all three data sets. Summary of diffraction data, refinement statistics and the quality indicators for the structures are given in Supplementary Material, Table S1.

### Oligomerisation of hippocalcin proteins

*Size Exclusion Chromatography Multiangle Laser Light Scattering (SEC-MALS).* Hippocalcin proteins (100 μl) were separated on a Superdex 200 10/300 GL gel filtration column (GE Healthcare Life Sciences) equilibrated in 50 mM HEPES (pH 7.5) containing either 1 mM CaCl_2_ or 5 mM EGTA at 0.75 ml/min (AKTA Pure, GE Healthcare). Protein elution was monitored by a Wyatt HELEOS-II 18-angle laser photometer (DAWN8+ for light scattering measurement, Wyatt Technology) and an Optilab rEX refractive index detector. Molar masses were obtained using the Astra software (version 6.1.2.84) (Wyatt Technology).

### Characterization of binding of biotinylated hippocalcin proteins to VGCCs protein fragments

P/Q- (residues 1898-2035), N-type (residues 1836-1983) fragments and SUMO (negative control) proteins were separated on SDS-PAGE (NuPAGE 4–12% Bis-Tris, NuPAGE MOPS SDS running buffer, Life Technologies) were blotted onto nitrocellulose membranes for analysis. Biotinylated BSA was added to the samples as loading control. Membranes were incubated in blocking solution (5% BSA in PBS, 0.2% Tween 20) at room temperature for 1 h and then incubated overnight at 4 °C with biotinylated probes at 5 μg/ml in 5% BSA in 50 mM Tris, 0.5 M NaCl, 0.5% Tween 20 (with or without the addition of 5 mM EGTA). Membranes were washed 3 times in 50 mM Tris, 0.5 M NaCl, 0.5% Tween 20 ± 5 mM EGTA and incubated with Streptavidin-HRP conjugate (Amersham) peroxidase at 1:400 in blocking solution for 1 h. The membranes were washed 3 times as above, and bound biotinylated proteins were detected using Pierce ECL western blotting substrate (Thermo Scientific) on a ChemiDoc XRS+ (Biorad).

### Calcium imaging of hippocalcin wild-type and mutants in differentiated SH-SY5Y cells

Differentiated SH-SY5Y cells were transfected with hippocalcin-mCherry constructs and loaded with 1 μM Fluo-4-AM (Molecular Probes) for 30 min in the absence or in the presence of 0.4 μM ω-agatoxin IVA (Abcam, P/Q-type calcium channel blocker), 10 μM ω-conotoxin MVIIC (Sigma, P/Q- and N-type calcium channel blocker) or 1 μM ω-conotoxin GVIA (Abcam, N-type calcium channel blocker). Cells were examined at 37 °C (OKO lab incubation chamber) in a 35-mm glass bottom dish (MatTek) with a 3i Marianas spinning-disk confocal microscope equipped with a Zeiss AxioObserver Z1, a 40x/1.3 oil immersion objective and a 3i Laserstack as excitation light source (488 nm, for Fluo-4; 561 nm, for hippocalcin-mCherry). Emitted light was collected through single bandpass filters (Yokogawa CSU-X filter wheel) onto a CMOS camera (Hamamatsu, ORCA Flash 4.0; 1152x1656 pixels).

Cells were stimulated with 50 mM KCl and images were collected every 5 s for 1 min. The time course for intracellular calcium influx was monitored over an elliptical region of interest (ROI) in the cell body using ImageJ program. Data obtained from 9 to 22 cells was plotted and analysed on GraphPad Prism.

### Data analysis and statistics

Results are expressed as mean ± SEM unless indicated otherwise. Biophysical experiments were performed at least in triplicates and analysed using GraphPad Prism software. Cell experiments were carried out on three independent preparations each. The total number of cells analysed in each condition is given in the figure legends. Significance level was obtained using Student *t*-test with the wild-type or untreated as control. *P* values in the figures and tables are represented by stars (*P < 0.05, **P < 0.01, ***P < 0.001, ****P < 0.0001, ns for non-significant).

## Supplementary Material

Supplementary Data
