# Supplementary material for: Biophysical and functional characterization of hippocalcin mutants responsible for human dystonia
Source: Hum Mol Genet. 2017 Apr 7;26(13):2426–35. doi: 10.1093/hmg/ddx133 (PMC5886089; doi:10.1093/hmg/ddx133)
Supplement: Supplementary Data [file ddx133_supp.pdf]

Biophysical and functional characterization of hippocalcin mutants responsible for human dystonia

Nordine Helassa[1*], Svetlana V. Antonyuk[2], Lu-Yun Lian[3], Lee P. Haynes[1] and Robert D. Burgoyne[1]

[1]Department of Cellular and Molecular Physiology, Institute of Translational Medicine, University of Liverpool, Liverpool L69 3BX, United Kingdom. [2]Molecular Biophysics Group, Institute of Integrative Biology, Faculty of Health and Life Sciences, University of Liverpool, Liverpool L69 7ZB, United Kingdom. [3]NMR Centre for Structural Biology, Institute of Integrative Biology, Faculty of Health and Life Sciences, University of Liverpool, Liverpool L69 7ZB, United Kingdom.

[*]Corresponding author: nhelassa@liverpool.ac.uk

Materials and Methods

Determination of hippocalcin proteins stability. *Trypsin limited proteolysis*. Biotinylated hippocalcin proteins (20 μg/ml) were incubated with trypsin (0.5 μg/ml) for 0, 5, 10, 15, 20 and 30 min at 37 °C. The reaction was stopped using 5 mM PMSF. Degradation of hippocalcin was quantified by western blot (streptavidin-HRP conjugate) using ImageJ software.

Heat denaturation. Heat stability of hippocalcin proteins was determined by following the unfolding of the α-helices at 222 nm using circular dichroism (JASCO J-1100 CD spectrometer, 200 μl quartz cuvette, 0.1 cm path length). Samples (15 μM) were measured in MilliQ water containing either 1 mM $CaCl_2$ or 5 mM EGTA. Data was collected from 20 to 90 °C using 1 °C increment and a ramp increase of 5°C/min.

Guanidine denaturation. Chemical stability of hippocalcin proteins was determined by measuring the intrinsic tryptophan fluorescence after guanidine denaturation (JASCO FP-6300 fluorimeter, 1 mL quartz cuvette). Proteins (~ 10 μM) in 50 mM K^+-HEPES, 100 mM KCl, 2 mM $MgCl_2$, pH 7.5, mixed with increasing concentration of guanidine ([Gnd-HCl] from 0 to 3 M). Proteins were excited at 280 nm and fluorescence emission spectra were collected from 290 to 410 nm.

Structure determination of hippocalcin proteins. *Circular dichroism (CD) spectroscopy*. CD spectra were recorded using a JASCO J-1100 spectrometer equipped with a JASCO MCB-100 mini circulation bath for temperature control. Far-UV CD spectra (180-260 nm) were recorded at 20 °C in a 0.1 cm path length quartz cell (5 accumulations, scan rate 50 $nm.min^{-1}$). Proteins (15 μM) were measured in MilliQ water containing either 1 mM $CaCl_2$ or 5 mM EGTA. Secondary structure content was determined using CDSSTR prediction algorithm (DichroWeb online server) (1, 2).

Oligomerisation of hippocalcin proteins. *In vitro crosslinking.* Hippocalcin purified proteins (10-20 μM) were crosslinked using 20-50 fold molar excess of DSP or BS3 crosslinker (ThermoScientific) in the presence of 0.05-0.1-0.5-1 mM $CaCl_2$ or 5 mM EGTA. The mixture was incubated at room temperature for 30 min and the reaction was quenched by the addition of 50 mM Tris for 10 min.

In vivo crosslinking. N2A cells were grown on a 6-well plate in DMEM supplemented with 5% fetal bovine serum, 1x non-essential amino-acids and 1% Penicillin-Streptomycin solution. At 90% confluence, cells were transfected using Lipofectamine™ 2000 (Invitrogen) following the manufacturer's recommendations. Crosslinking was performed 24 hours post-transfection by washing the cells with PBS and incubated with 2 mM DSP for 30 min. After the reaction was stopped by 50 mM Tris for 10 min, the cells were lysed in RIPA buffer for 30 min at 4°C. Clarified lysates were loaded on SDS-PAGE (NuPAGE 4-12% Bis-Tris, NuPAGE MOPS SDS running buffer, Life Technologies) with or without pre-incubation with a reducing agent to cleave the crosslinker (50 mM DTT for 30 min at 37°C), for Western blot analysis using anti-GFP monoclonal antibody (Roche) and densitometry quantification.

Translocation experiment using photo-release/confocal imaging. Hippocalcin exhibits a Ca^{2+}/myristoyl switch mechanism which allow the protein to remain cytosolic at resting calcium and to translocate to cellular membranes when intracellular

calcium concentration increases (10). SH-SY5Y cells transfected with hippocalcin-mCherry constructs were loaded with 1 μM Fluo-4-AM (Molecular Probes) and 25 μM NP-EGTA (Molecular Probes) and incubated in growth medium for 30 min at 37 °C. Cells were examined in a 35-mm glass bottom dish (MatTek) with a 3i Marianas spinning-disk confocal microscope equipped with a Zeiss AxioObserver Z1, a 63x/1.4 oil immersion objective and a 3i Laserstack as excitation light source (405 nm, for NP-EGTA photolysis; 488 nm, for Fluo-4; 561 nm, for hippocalcin-mCherry). Emitted light was collected through single bandpass filters (Yokogawa CSU-X filter wheel) onto a CMOS camera (Hamamatsu, ORCA Flash 4.0; 1152x1656 pixels). Experiments were carried out at 37 °C (OKO lab incubation chamber) and images were collected at 2 frames/s. To investigate the calcium-dependent translocation of hippocalcin, photolysis of NP-EGTA was performed by illumination with 405-nm laser light at 10% power for 1 ms for rapid release of [Ca^{2+}]. The time course for intracellular calcium release and hippocalcin translocation after photo-stimulation was monitored at the *trans*-Golgi network (TGN) over an elliptical region of interest (ROI). The mean background fluorescence was measured by ROI with the same dimensions placed at a point at which no cell was present. Fluo-4 and hippocalcin fluorescence changes were obtained from the background-corrected fluorescence with ImageJ program. Data obtained from 10 cells was normalised and plotted on GraphPad.

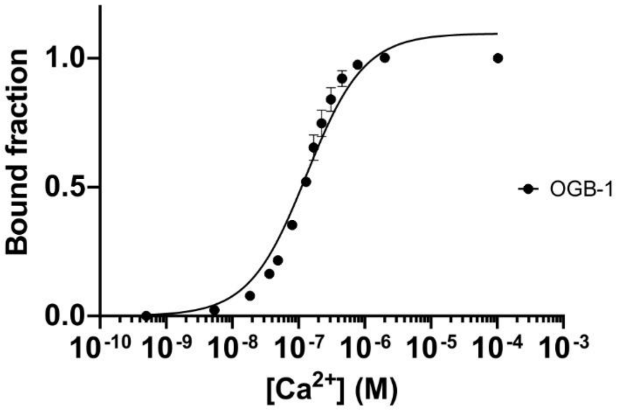

Figure S1. Verification of [Ca^{2+}] used for hippocalcin equilibrium titrations with OGB-1. A K_d value of 132 ± 11 nM was obtained, not significantly different from reported by Molecular Probes (170 μM in buffer without magnesium).

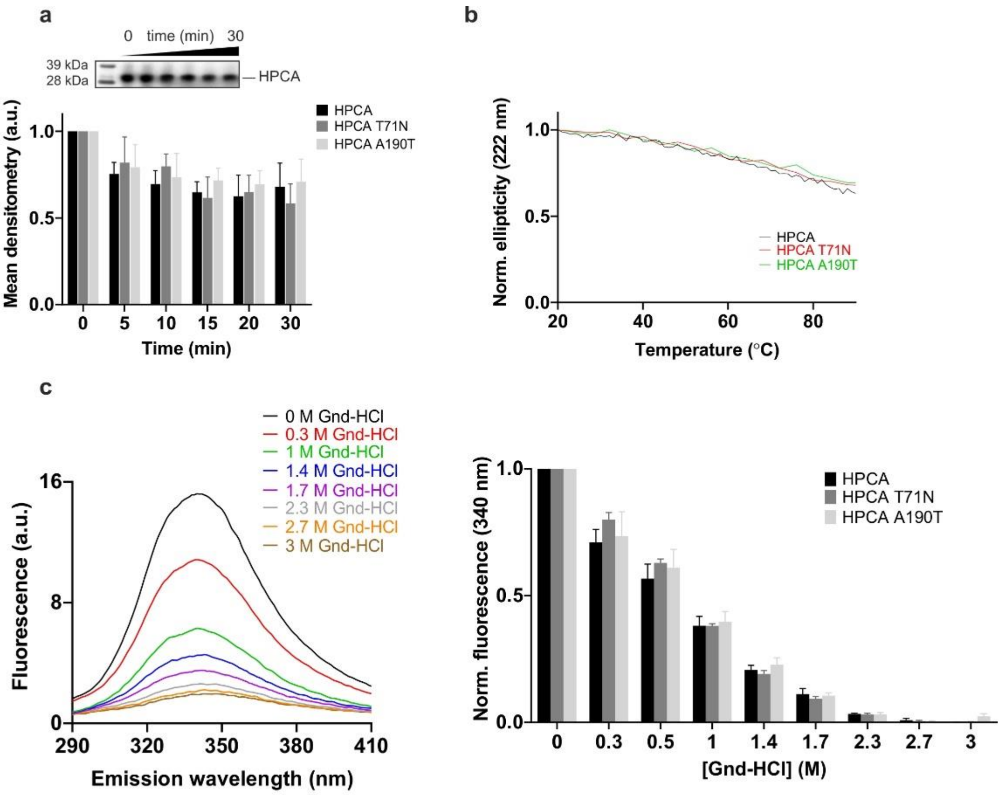

Figure S2. Effect of dystonia-causing mutations on trypsin, temperature and chemical susceptibility of hippocalcin. (a) Trypsin limited proteolysis of hippocalcin wild-type and mutants. Western blots of purified hippocalcin proteins were incubated with trypsin for 0, 5, 10, 15, 20 and 30 minutes at 37 °C. Bands were quantified by densitometry using ImageJ software. (b) Thermal unfolding of hippocalcin wild-type and mutants monitored by circular dichroism recorded at 222 nm from 20 to 90 °C. (c) Typical tryptophan emission spectra of hippocalcin and denaturation-induced fluorescence of hippocalcin wild-type and mutants as a function of [Gnd-HCl] (left panel). Quantification of the emission fluorescence at 340 nm as a function of Gnd-HCl concentration did not show any significant difference between the wild-type and the mutants (right panel).

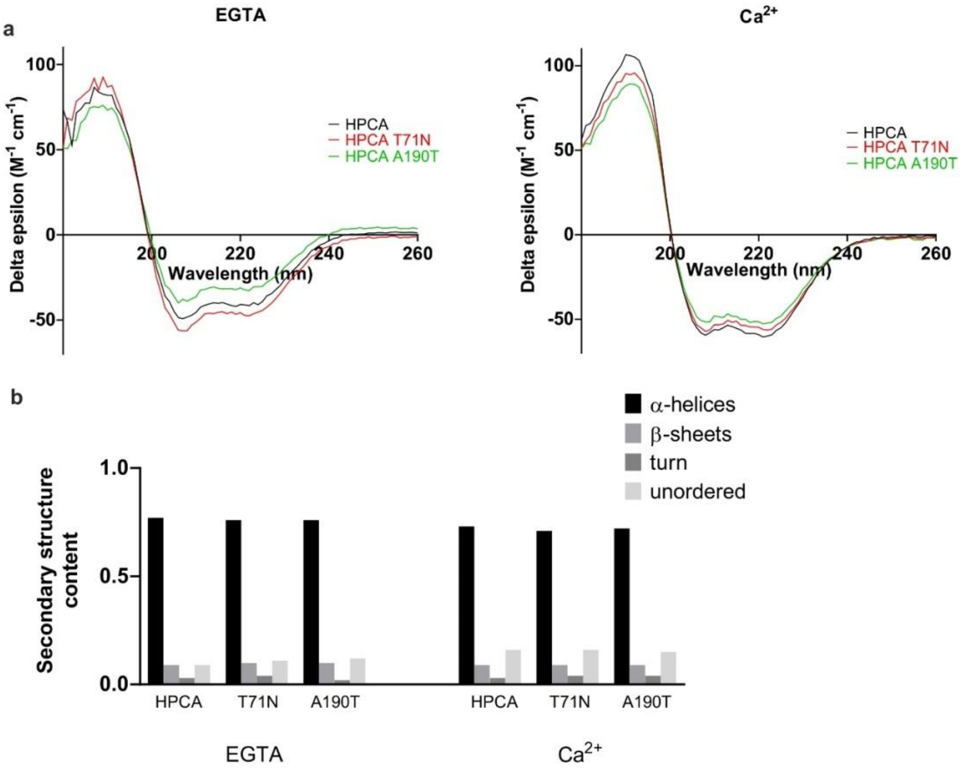

Figure S3. Analysis of the secondary structures of hippocalcin wild-type and mutants using circular dichroism (CD) spectroscopy. (a) CD spectra were obtained in the presence of 5 mM EGTA (left panel) or 1 mM $CaCl_2$ (right panel) and (b) protein secondary structure content was estimated using CDSSTR method (Johnson (1999); Whitmore and Wallace (2004)) showing no significant difference between hippocalcin wild-type and the mutants.

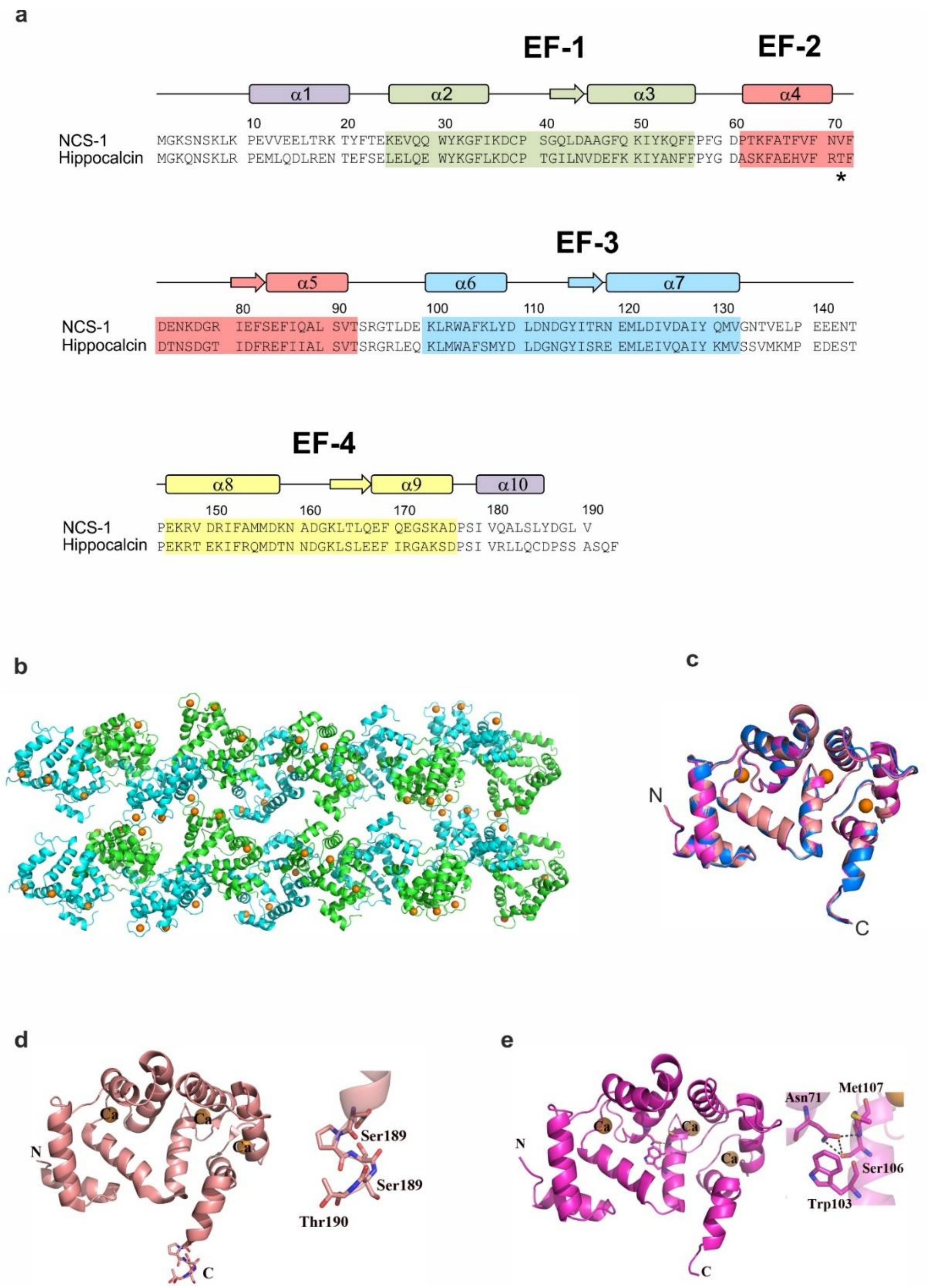

Figure S4. Secondary and crystal structure of hippocalcin, hippocalcin(T71N) and hippocalcin(A190T). (a) Amino-acid and secondary structure alignment of full-length human hippocalcin and rat NCS-1. Positions of the dystonia-causing mutations are shown with a star. (b) Cartoon representation of the crystal packing of hippocalcin showing the long parallel protein fibrils morphology. (c) Alignment of hippocalcin crystal structure (magenta) with hippocalcin(T71N) (marine) and hippocalcin(A190T) (salmon) did not show any significant difference. Cartoon representation of the (d) hippocalcin(A190T) and (e) hippocalcin(T71N) structure. Details of the mutation site are shown as sticks. C-terminus is labelled with letter C, N-terminus – N. Ca^{2+} ions in EF-2, EF-3 and EF-4 are shown as orange spheres.

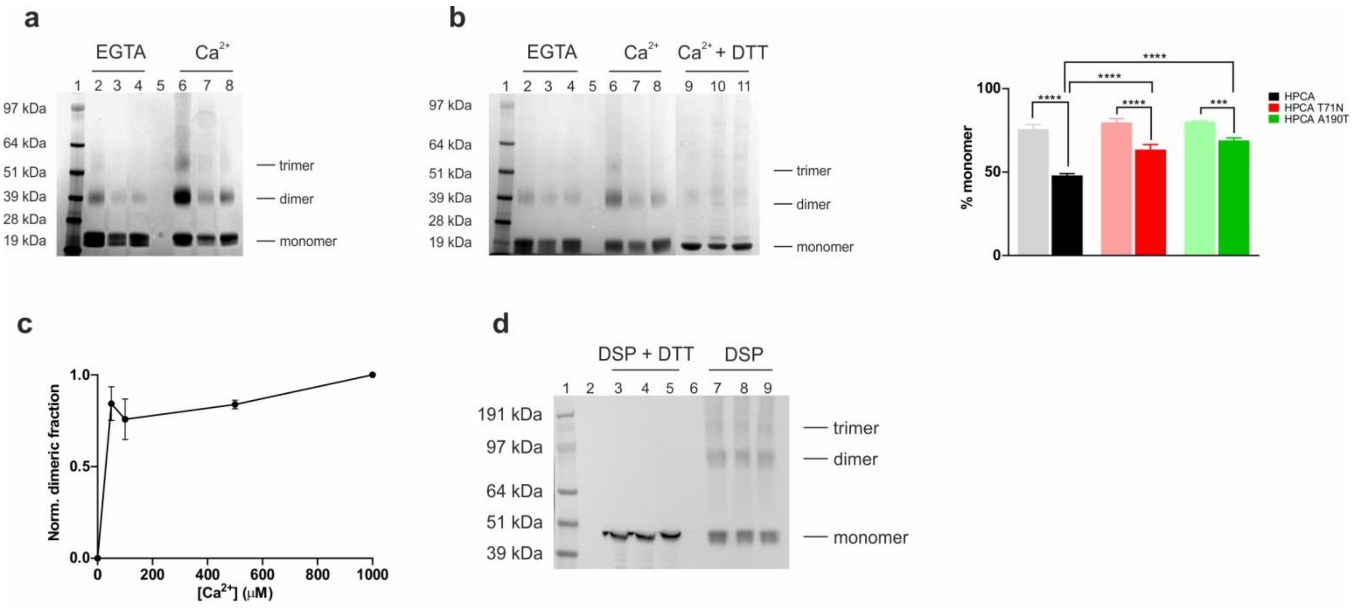

Figure S5. Calcium-dependent oligomerisation of hippocalcin. (a) SDS-PAGE of hippocalcin purified proteins after *in vitro* crosslinking using BS3 crosslinker showing oligomer formation in the presence of calcium. Lane 1: molecular weight ladder, lane 2,6: hippocalcin wild-type, lane 3,7: hippocalcin(T71N), lane 4,8: hippocalcin(A190T), lane 2-4: in the presence of 5 mM EGTA, lane 6-8: in the presence of 1 mM $CaCl_2$. (b) SDS-PAGE of hippocalcin purified proteins after *in vitro* crosslinking using DSP crosslinker showing oligomer formation in the presence of calcium. Lane 1: molecular weight ladder, lane 2,6,9: hippocalcin wild-type, lane 3,7,10: hippocalcin(T71N), lane 4,8,11: hippocalcin(A190T), lane 2-4: in the presence of 5 mM EGTA, lane 6-8: in the presence of 1 mM $CaCl_2$, lane 9-11: in the presence of 1 mM $CaCl_2$ and 50 mM DTT (left panel). Bands were quantified by densitometry using ImageJ (right panel). Light colors in the absence of calcium, dark colors in the presence of calcium. (c) Dimeric fraction of hippocalcin wild-type purified protein after *in vitro* crosslinking using DSP at $[Ca^{2+}]$ of 50 μM, 100 μM, 500 μM and 1 mM. Crosslink reactions were run on SDS-PAGE and analysed by densitometry using ImageJ. Data were normalised to the dimeric fraction obtained at 1 mM $[Ca^{2+}]$ and showed that 50 μM $[Ca^{2+}]$ was sufficient to induce oligomer formation. (d) Western blot of *in vivo* crosslinking of N2A cells transfected with EGFP-hippocalcin using DSP showing that oligomerisation occurs at physiological conditions. Lane 1: molecular weight ladder, lane 3,7: hippocalcin wild-type, lane 4,8: hippocalcin(T71N), lane 5,9: hippocalcin(A190T), lane 3-5: in the presence of 50 mM DTT, lane 7-9: in the absence of DTT.

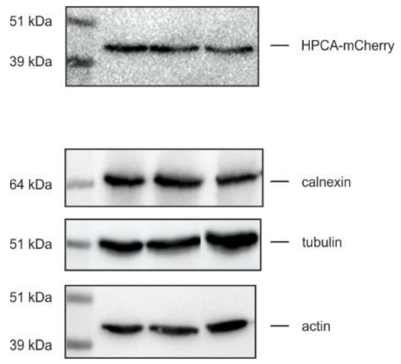

Figure S6. Expression level of transfected SH-SY5Y cells with mCherry tagged hippocalcin (RFP antibody). Lane 1: molecular weight ladder, lane 2: hippocalcin wild-type, lane 3: hippocalcin(T71N), lane 4: hippocalcin(A190T). Calnexin, tubulin and actin antibodies used as loading controls showed that expression levels were similar for all hippocalcin proteins.

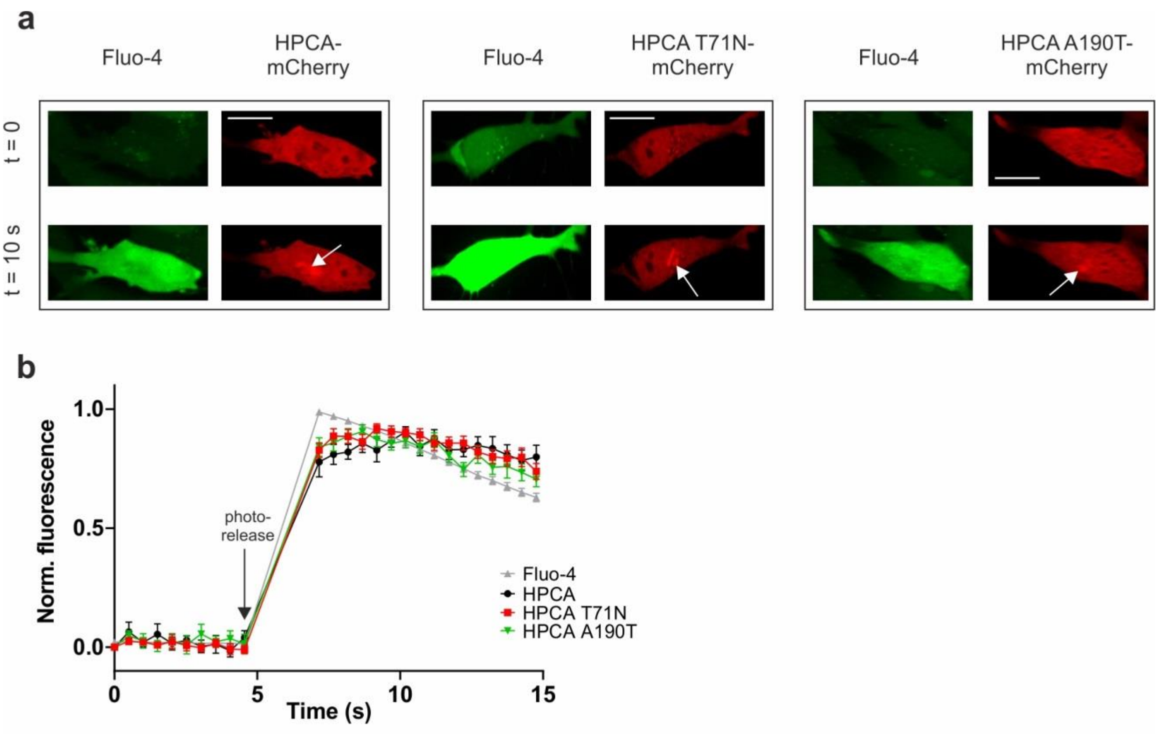

Figure S7. Rapid translocation of hippocalcin wild-type and dystonia-causing mutants after flash photolysis of NP-EGTA. SH-SY5Y cells transfected with hippocalcin-mCherry constructs were loaded with the calcium dye Fluo-4 and the calcium chelator NP-EGTA. After rapid calcium photorelease, live cells were imaged on a spinning-disk confocal microscope (n = 30). (a) Representative microscopy images before and after calcium photorelease revealed no significant alteration of translocation properties. Arrows indicate the location of the translocated hippocalcin after calcium photorelease. (b) Time course of intracellular calcium increase followed by Fluo-4 and hippocalcin translocation, showing that translocation occurs when intracellular calcium increases. Scale bar = 10 μm.

Table S1. Data collection and refinement statistics.

Values in parentheses are for the highest resolution shell.

	HPCA	HPCA A190T	HPCA T71N
Data collection Wavelength (Å)	0.98	0.92	0.98
Beamline	IO2	IO4	IO4-1
Detector	Pilatus	Pilatus	Pilatus
Space group	$P6_1$	$P6_1$	$P6_1$
Unit-cell dimensions (a,b,c) (Å)	50.91, 50.91, 283.02	50.66, 50.66, 283.46	51.16, 51.16, 284.06
Resolution (Å)	43.56-2.42 (2.59-2.42)	43.87-2.54 (2.6-2.54)	47.3-3.00 (3.18-3.00)
Rmerge % (last shell)	4.4 (63.0)	8.6(68)	12.8(57)
I/σ (last shell)	11.7 (1.8)	8.7(1.9)	4.9(1.6)
Completeness (%)	98.8 (99.9)	99.7(99.6)	99.0(99.0)
Redundancy	3.3 (3.2)	4.6(4.4)	2.7(2.7)
Wilson B factor ($Å^2$)	62	56	62
No. of reflections	15734	13592	8283
Rwork/Rfree	23.3/27.1	21.9/24.9	21.9/28.3
No. of atoms			
Protein	2996	2996	3021
Ca ions	6	6	6
Water	34	34	22
B factor ($Å^2$)			
Protein	57.8	55	78
Ca^{2+} ions EF-2	75	73	83
Ca^{2+} ions EF-3	83	77	85
Ca^{2+} ions EF-4	105	71	78
Waters	65	50	47
R.M.S deviations			
Bond length (Å)	0.014	0.012	0.008
Bond angles (°)	1.98	1.5	1.14
PDB code	5g4p	5g58	5m6c

Table S2. Molar mass calculated from MALS data for HPCA and HPCA mutants in the presence or absence of calcium

	**Molar mass (kDa)** in the presence of 5 mM EGTA				**Molar mass (kDa)** in the presence of 1 mM $CaCl_2$			
	Peak 1	Peak 2	Peak 3	Peak 4	Peak 1	Peak 2	Peak 3	Peak 4
**HPCA**	23.1 ± 0.2	45.5 ± 3.1	-	-	25.1 ± 0.9	46.7 ± 1.1	68.2 ± 1.9	91.6 ± 2.9
**HPCA T71N**	23.4 ± 0.3	34.5 ± 8.1	-	-	24.1 ± 0.8	47.0 ± 0.2	65.7 ± 2.3	-
**HPCA N75K**	23.0 ± 0.2	-	-	-	22.5 ± 0.9	42.8 ± 1.9	65.9 ± 1.9	-
**HPCA A190T**	22.9 ± 0.2	44.8 ± 0.5	-	-	23.8 ± 0.2	44.5 ± 1.1	63.9 ± 0.6	-
